# Normalized Lift: An Energy Interpretation of the Lift Coefficient Simplifies Comparisons of the Lifting Ability of Rotating and Flapping Surfaces

**DOI:** 10.1371/journal.pone.0036732

**Published:** 2012-05-21

**Authors:** Phillip Burgers, David E. Alexander

**Affiliations:** 1 Department of Arts and Sciences, Embry Riddle Aeronautical University, San Diego, California, United States of America; 2 Department of Ecology and Evolutionary Biology, University of Kansas, Lawrence, Kansas, United States of America; University of Zurich, Switzerland

## Abstract

For a century, researchers have used the standard lift coefficient *C_L_* to evaluate the lift, *L*, generated by fixed wings over an area *S* against dynamic pressure, *½ρv* ^2^, where *v* is the effective velocity of the wing. Because the lift coefficient was developed initially for fixed wings in steady flow, its application to other lifting systems requires either simplifying assumptions or complex adjustments as is the case for flapping wings and rotating cylinders.

This paper interprets the standard lift coefficient of a fixed wing slightly differently, as the work exerted by the wing on the surrounding flow field (*L/ρ·S*), compared against the total kinetic energy required for generating said lift, *½v^2^*. This reinterpreted coefficient, the normalized lift, is derived from the work-energy theorem and compares the lifting capabilities of dissimilar lift systems on a similar energy footing. The normalized lift is the same as the standard lift coefficient for fixed wings, but differs for wings with more complex motions; it also accounts for such complex motions explicitly and without complex modifications or adjustments. We compare the normalized lift with the previously-reported values of lift coefficient for a rotating cylinder in Magnus effect, a bat during hovering and forward flight, and a hovering dipteran.

The maximum standard lift coefficient for a fixed wing without flaps in steady flow is around 1.5, yet for a rotating cylinder it may exceed 9.0, a value that implies that a rotating cylinder generates nearly 6 times the maximum lift of a wing. The maximum normalized lift for a rotating cylinder is 1.5. We suggest that the normalized lift can be used to evaluate propellers, rotors, flapping wings of animals and micro air vehicles, and underwater thrust-generating fins in the same way the lift coefficient is currently used to evaluate fixed wings.

## Introduction

The lift coefficient, as currently defined for fixed wings, has been successfully used in aerodynamics for almost a century. Aviation pioneer Otto Lilienthal was the first to use a form of dimensionless coefficient in equations for lift and drag, but the lift coefficient in its standard form was developed by Ludwig Prandtl around the time of the First World War, and first published in English in 1923 [1]. The lift coefficient, initially applied only to fixed wings, compares the wing loading—the lift force *L* distributed over a wing surface *S*—against a benchmark, the dynamic pressure, *½ρv^2^*. The lift coefficient as used today (hereinafter referred to as the standard lift coefficient, *C_L_*) is given by:
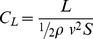
(1)where *L* is lift, *ρ* is air density, *v* is oncoming or effective air speed, and *S* is the wing area. (See Appendix S1 for a list of all symbols used.) The above definition of lift coefficient is used for steady and quasi-steady flight analysis. When using this equation for flapping flight, which involves quasi-steady aerodynamics, the effective velocity *v* of the wing relative to the surrounding flow field is calculated by a time-dependent series of steady state flow cases over the static wing at appropriate intervals of static angles of attack. The resultant time-dependent lift is then summed along the wing area through the wing beat cycle. The mean lift coefficient is then calculated dividing the resultant lift by the product of an “effective dynamic pressure” and a reference area.

Because the *C_L_* was developed initially for fixed wings in steady flow, its application to other lifting structures (e.g., flapping wings, rotating seeds) requires either simplifying assumptions or complex adjustments (e.g., [2]). Recent studies have successfully related flapping, spinning and translating by using dimensionless coefficients and using the dynamic pressure as the benchmark pressure [3]. This paper presents a new interpretation of the standard lift coefficient that evaluates and compares the ability of dissimilar lift systems to generate lift.

This paper proposes a dimensionless coefficient that compares the ability of dissimilar lift systems to generate lift by evaluating them on a similar footing. We show (1) that the standard lift coefficient of a fixed wing can be interpreted as the work per unit mass performed by the fixed wing on the surrounding flow field (*L/ρ⋅S*) normalized by the *specific kinetic energy* (or kinetic energy per unit mass) of the fixed wing and (2) that this interpretation can be applied directly to rotating cylinders and spheres and flapping wings.

## Methods

The standard lift coefficient for fixed wings can be interpreted as a ratio of the cost of generating work per unit mass on the surrounding flow field, *L/ρ⋅S*, with a benchmark, the total kinetic energy per unit mass of the wing, *½v^2^*. A typical maximum lift coefficient of 1.5 measured for a fixed wing without high lift devices can be interpreted as the specific work exerted on the surrounding flow field that is 50% higher than the specific kinetic energy possessed by the fixed wing. This new interpretation of the lift coefficient as a ratio of the specific work on the flow field over total specific kinetic energy of the wing breaks down when applied to rotating cylinders and flapping wings because the denominator, *½v^2^*, only accounts for the forward speed of these other lift systems, and does not account for the available specific kinetic energy due to the rotation of the cylinder or the flapping of wings that contributes to the generation of lift. In other words, the product *½v^2^* represents the total specific kinetic of a fixed wing, but this same energy benchmark is still used for rotating cylinders and spheres and flapping wings. For this reason, the standard lift coefficient overestimates the ability to generate lift by flapping wings and rotating cylinders. The total specific kinetic energy of a flapping wing in forward flight or a rotating cylinder in Magnus effect is higher than *½v^2^*, and so, this term, which is the specific kinetic energy due to the forward speed of a fixed wing, must be increased by the specific kinetic energy due to the flapping of the wings or to the rotation of the cylinder.

Each lifting surface, whether fixed, flapping or rotating, has its own characteristic energy benchmark. Extending the energy interpretation of the standard lift coefficient of fixed wings to flapping and rotating lifting mechanisms, the energy benchmark for a given lifting surface is then the total specific kinetic energy of the lifting surface while it generates lift (see Table 1). We suggest that the best way to evaluate the ability of a lifting system to generate lift is the ratio of the work done by the lifting surface and its corresponding total specific kinetic energy. For fixed wings, this ratio equals the standard lift coefficient, but for rotating or flapping systems, the denominator should include the specific kinetic energy available due to rotation for cylinders and spheres or due to flapping for flapping wings, and we call this ratio the *normalized lift*.

**Table 1:** Normalized lift *L_N_* equations for different lift-generating systems.

Type of Lift Generation	Normalized Lift (*L_N_*)
Fixed wing	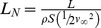
Rotating cylinder (Flettner rotor)	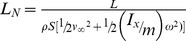
Flapping wing, hovering	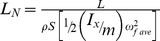
Flapping wing in forward flight	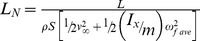
Flapping wing in forward flight, with pronation and supination	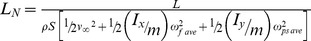

The derivation of normalized lift is based on the work-energy theorem in which the energy benchmark is calculated by adding algebraically each of the scalar, specific energy terms that contribute to the generation of lift. The term *½v^2^* from the denominator of equation 1 is the total specific kinetic energy for a fixed wing and can be interpreted as either contained in the fluid moving toward a fixed wing or in the fixed wing moving through the still fluid. Because specific energy is a scalar value, if a lifting system differs from a fixed wing and has other sources of kinetic energy, additional terms for those other sources can simply be added to form the benchmark.

The work-energy theorem states that the work done on a system equals the increase in kinetic energy of the system [4]. In an ideal, no-friction, one-dimensional case, the ratio of work by a constant force, *F*, exerted on an object displaced a distance, *d*, and the resulting change in total kinetic energy is given by:
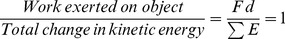
(2)where *E* equals kinetic energy. Equation 2 assumes that the object is not self-propelled or experiencing friction when subjected to external work. If the object is self-propelled or it experiences friction, equation 2 may take values somewhat lower or higher than one.

We now apply the work-energy theorem to the lift force, *L*, acting on a three-dimensional parcel of inviscid, incompressible fluid. Substituting *L* for the force *F*, and dividing both the numerator and denominator by the mass of the fluid (*ρ* 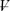 gives:
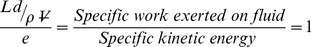
(3)This expression of the work-energy theorem states that the specific work exerted by a lifting surface on a parcel of volume *V* of inviscid, incompressible fluid of density *ρ* as it is displaced a distance *d*, increases the specific kinetic energy *e* of the fluid around it. As mentioned above, the interchangeability between the total specific kinetic energy of the system generating lift and the surrounding flow field permits us to calculate the total specific kinetic energy interchangeably with a lifting surface moving through a fluid or by the fluid flowing over the lifting surface. This allows calculating the denominator of equation 3 by calculating the total specific kinetic energy of the rotating cylinder or the flapping wing, rather than calculating the kinetic energy of the surrounding flow field. Thus, the energy benchmark used in the calculation of normalized lift, the total specific kinetic energy of the lifting surface, is obtained by simply adding algebraically the various types of specific kinetic energy of the lifting surface under study.

The inverse of the ratio *d/* 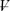 in equation 3 represents the reference area, *S*. For flapping morphing wings some researchers use non-dimensional drag and lift coefficients that include the surface area effect [5]. In this paper, the reference area *S* is the planform area of the wing. We define the normalized lift for any lifting surface as the ratio of specific work on the fluid to this energy benchmark, the total specific kinetic energy possessed by that lifting surface. Thus, for a fixed wing,
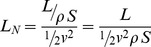
(4)which is mathematically the same as the standard lift coefficient. As the flyer may be self- propelled and is immersed in a viscous fluid, the ratio in equation 4 may no longer be exactly 1 as stated by the work-energy theorem. The term “normalized” makes explicit the fact that the comparison of the cost of generating lift by fixed wings, rotating cylinders and flapping wings are now compared with respect to the corresponding total specific kinetic energy, instead of using a common benchmark, that of the fixed wing. The derivation of the normalized lift using the work-energy theorem gives this coefficient a direct connection to its physical basis.

The normalized lift of a wide variety of lifting surfaces can be calculated by adding a summation to the denominator of equation 4 that contains as many *n* terms as sources of kinetic energies possessed by the lifting surface (e.g., translational, rotational, flapping, flutter, pronation and supination). For example, a cylinder experiencing the Magnus effect (such as a Flettner rotor) has a kinetic energy term dependent on the translational speed equivalent to the far field speed *v_∞_* that is not perturbed by the cylinder's rotation. The cylinder also has a kinetic energy term dependent on the rotational speed that defines the cylinder's near flow field speed *u_nf_*, thus giving the cylinder a second source of kinetic energy that contributes to the generation of lift. To capture the effects of these various types of specific kinetic energy, we can rewrite equation 4 to define the general form of the normalized lift, *L_N_*, as:
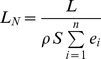
(5)To calculate the normalized lift of a flapping bird with forward speed, we calculate the total specific kinetic energy of the wings by simply adding the specific kinetic energy due to the translational speed *v_∞_* of the lifting surface, and the intrinsic specific kinetic energy due to flapping. These terms are shown in brackets below:
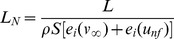
(6)The terms in brackets in the denominator of eq. 6 represent the sum of the specific kinetic energy, *e_i_(v_∞_)*, a function of the far field speed *v_∞_* due to forward velocity, and the specific kinetic energy, *e_i_(u_nf_)*, a function of the near field *u_nf_* due to the lifting surface's flapping, rotation, etc. The difference between the standard lift coefficient, *C_L_*, and the normalized lift *L_N_*, is that the normalized lift accounts for all the specific kinetic energies involved in the generation of lift, whereas the standard *C_L_* only considers the kinetic energy due to forward speed and does not account for the energy sources due to flapping, rotation, etc. For this reason, the maximum lift coefficient of a rotating cylinder can be nearly 7 times greater than for a fixed wing. We believe that it is at this point that the lift coefficient breaks down in its task of comparing the ability of dissimilar lift systems to generate lift. We note that some recent authors have used the standard lift coefficient with a more precise effective velocity for the flapping wing that have yielded less extreme lift coefficient values [6].

When equation 6 is applied to a fixed wing aircraft flying straight and level with only translational kinetic energy, the *e_i_(v_∞_)* term in the denominator is ½*v_∞_* ^2^ and the *e_i_(u_nf_)* term is zero as the wing does not flap or rotate; thus, the normalized lift, *L_N_*, equals the standard lift coefficient *C_L_*. If the lifting surface flaps or rotates, equation 6 can be applied directly by adding the appropriate *e_i_(u_nf_)* terms, whereas the traditional lift coefficient will need extensive modification to account for the complex effective velocity [2], [7], [8]. Thus, the normalized lift for hovering insects and birds finds only the term *e_i_(u_nf_)* in the brackets of equation 6 whereas *e_i_(v_∞_)* is zero. See the case for flapping wing, hovering in Table 1.

This lift normalization allows the comparison of lifting capabilities of a large variety of lifting systems on the same energy terms. A rotating cylinder moving through a fluid at right angles to its long axis (a Flettner rotor) produces lift via the Magnus effect [9], and the standard lift coefficient for such a cylinder can exceed 9.0 [10]. Similarly, *C_L_* values for the flapping wings of flying animals have been reported ranging from >4.0 for true flies [7] to >5.0 for a small bird [8]. Such high standard lift coefficients seem to indicate that a flapping wing or a rotating cylinder is many times more effective at producing lift than a simple fixed wing. While these very high maximum lift coefficients may indicate differences in how these devices produce lift, the values of the coefficients themselves may be misleading when comparing the costs of lift production.

## Results

We illustrate the concept of normalized lift by calculating it for a gliding wing, a rotating cylinder, and a variety of flapping wings. Unlike the standard lift coefficient, the equation for normalized lift *L_N_* does not require any modification in order to be applied to more complex lifting systems than fixed wings.

### Application to a glider

The total specific kinetic energy of a glider that has two velocities defined at infinity, namely a horizontal speed component *v_h_*, and a vertical speed component *v_v_* (or sink speed), is
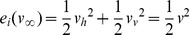
(7)Adding the horizontal and vertical speed components results in the absolute velocity of the glider *v*. Substituting the results of this summation into eq. 6, we obtain the normalized lift, *L_N_*:
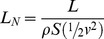
(8)Note that the normalized lift in this case (eq. 8) equals the traditional lift coefficient *C_L_* (eq. 1), which we interpret as the ratio of the work done by the fixed wing on the surrounding flow field to the energy benchmark, its total specific kinetic energy. This benchmark is the sum of the all the specific energies contributing to the generation of lift. The normalized lift coefficient, as is stated in eq. 8, illustrates the interchangeability between the kinetic energy *½v^2^* of the moving air flowing over an inert glider in a wind tunnel—a Lagrangian viewpoint—or the kinetic energy *½v^2^* of the glider flying through static air—an Eulerian viewpoint.

### Application to a rotating cylinder (Flettner rotor)

**Figure 1:**
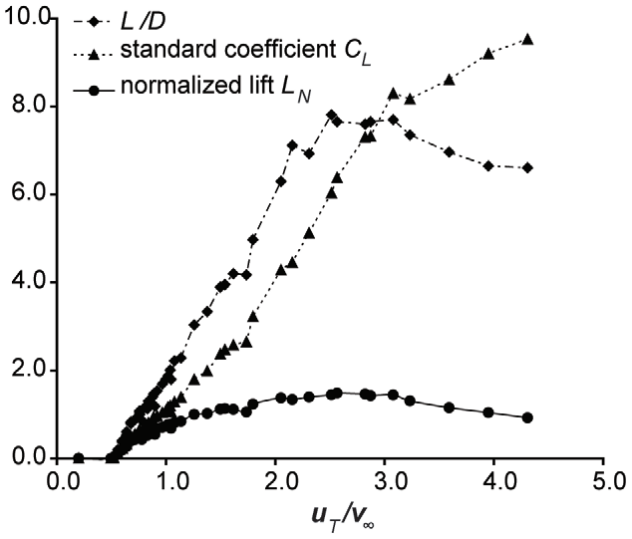
Coefficients for a rotating cylinder. Lift to drag ratio, *L/D*, standard lift coefficient, *C_L_*, and normalized lift, *L_N_*, (all dimensionless ratios), plotted against spin parameter (*u_T_/v_∞_*) for a rotating cylinder in Magnus effect. Data from Reid (1924).

A number of samaras (winged seeds) produce lift using the Magnus effect, operating as Flettner rotors [9], [11]. For a rotating cylinder with forward velocity *v_∞_* and angular velocity *ω*, the total kinetic energy of the cylinder equals
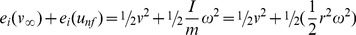
(9)We obtain the last term by replacing the moment of inertia *I* of the cylinder by *½mr^2^*. (See eq. S2.1 in Appendix S2 for detailed derivation.) Substituting this sum into eq. 6 and entering the experimental values of lift generated by a rotating cylinder [10], we can calculate the cylinder's normalized lift *L_N_* and compare them with the cylinder's standard *C_L_*. The reference area S of the cylinder is the same as the reference area for its standard lift coefficient, that is, the cross section perpendicular to the flow [10], [12]. Figure 1 shows the standard lift coefficient, *C_L_*, the normalized lift, *L_N_*, and lift to drag ratio, *L/D*, calculated from experimental lift values. They are plotted, as customary for Flettner rotors, against the spin parameter, which equals the ratio of tangential velocity to incoming (horizontal) velocity, *u_T_*/*v*. The behavior of the standard lift coefficient and normalized lift are quite different. The standard lift coefficient increases continuously over the whole range of measured spin parameters, reaching values over 9.0, even though the *L/D* peaks at *u_T_*/*v* = 2.5 and decreases at higher spin parameter values. In contrast, the *L_N_* never exceeds a value of 1.5, and like the L/D, it shows a peak very near *u_T_*/*v* = 2.5. The decrease in L/D at *u_T_*/*v*>2.5 probably indicates stall and large separation. This condition is not captured by the standard *C_L_*.

### Flapping example 1: Hovering bat

Hovering flight is particularly interesting for purposes of comparing the standard lift coefficient and the normalized lift for at least two reasons. First, the lack of horizontal airspeed in hovering actually simplifies the equation for *L_N_*, whereas using the standard *C_L_* requires extensive modification and additional complexity to obtain the effective velocity, *v* [2], [13]. Second, anomalously high values for lift coefficients compared to fixed wings have been reported for hovering animals, in some cases exceeding 5 or 6 (e.g., [8]).

To calculate the normalized lift *L_N_* for a flapping flyer, we determine the total kinetic energy due to flapping as shown for the case of flapping wing, hovering in Table 1. Because the animal is hovering, the kinetic energy term due to translation, *½v_∞_^2^*, is zero, so the total kinetic energy is due entirely to the wing's angular motion. In this case, we will only consider the angular dorsoventral movements of flapping (see eq. S2.2 in Appendix S2 for detailed derivation).

Using morphometric data and average values for flapping frequency and stroke angles given by Norberg [8] for a hovering long-eared bat, *Plecotus auritus* (Linnaeus), and using the total kinetic energy for flapping wings, we calculate *L_N_* = 1.03 (Table 2). This *L_N_* value is in marked contrast to the standard *C_L_* values of 3.1 to 6.4 originally reported. In contrast to the scalar simplicity of specifying the kinetic energy of the flapping wing, the standard lift coefficient deals with the three-dimensional vectorial complexity of the wing's effective velocity, including the instantaneous resultant of the speed of the flapping wing and the induced velocity by the wing on the surrounding air flow with its accompanying simplifying assumptions, such as a constant and uniform downward acceleration distributed around a 360° disc with the wing span as its diameter.

**Table 2:** Morphometric data, reported standard lift coefficients, and calculated normalized lift values (air density is assumed to be 1.2 kg m^−3^).

Species	Weight (N)	S (m^2^)	v (m s^−1^)	r (m)	*ϕ_f_* (°)	*f_f_* (s^−1^)	*c_ave_* *	*ϕ_ps_* (°)*	*C_L_*	*L_N_*
*Plecotus auritus* 1 (long-eared bat, hovering)	0.0883	0.0123	0	0.1240	120	11.4			3.1–6.4	1.03
*Glossophaga soricina* (bat 1)^2^ (long-tongued bat, forward flight)	0.104	0.00884	1	0.1185	65	16.7			≈10	2.55
			6.5		87	13.4			1.7	0.369
*G. soricina* (bat 2)^2^	0.107	0.00936	1	0.1215	65	17.6			≈10	2.17
			6.5		87	14.3			1.7	0.369
*Bibio marci* (march fly, hovering)^3^	0.00064	0.0000752	0	0.0112	139	99			4.42	1.473
							0.00335	60	4.42	1.467
							0.00335	120	4.42	1.449

* Note that *c_ave_* and *ϕ_ps_* values are only needed when pronation and supination are included in the normalized lift calculation.

Sources:

1 [8];

2 [14];

3 [7].

### Flapping example 2: Bat in forward flight

Wolf et al. [14] describe the kinematics and vortex wake of two specimens of the long-tongued bat, *Glossophaga soricina*, over a range of flight speeds. They estimated the standard lift coefficient based on total circulation, and reported standard *C_L_* of approximately 10 at 1.0 m s^−1^ and standard *C_L_* = 1.7 at a flight speed of 6.5 m s^−1^. Based on the morphological and kinematic data of the bat specimens of Wolf et al. [14], we calculate a mean *L_N_* of 2.36 at a flight speed of 1.0 m s^−1^ and *L_N_* = 0.38 at 6.5 m s^−1^ (Table 2). Thus, both the high speed and low speed *L_N_* values are substantially lower than the standard *C_L_* values.

### Flapping example 3: Hovering dipteran

In the preceding examples, we accounted for the intrinsic kinetic energy due to the angular motion of flapping. We did not, however, take into consideration kinetic energy due to pronation and supination (i.e., changes in wing incidence relative to the animal's longitudinal axis). Smaller animals or animals flying slowly tend to have larger changes in this pronation-supination (p-s) angle [15], so we now look at how p-s movements affect the *L_N_* for a hovering insect.

We have calculated *L_N_* for a hovering march fly (*Bibio marci* Linnaeus) based on dimensions and flight data from Ennos [7]. Using a modified form of the standard *C_L_*, Ennos calculated *C_L_* = 4.42 for this fly. Because the fly was hovering, the kinetic energy due to translation *½v_∞_^2^* again goes to zero, and we are left with terms due to dorsoventral flapping and p-s movements (see eq. S2.3 in Appendix S2 for detailed derivation). In Table 2, we give three *L_N_* values for this fly, the first using only the flapping term, and two others using both the flapping term and the p-s term. The second *L_N_* value includes the p-s term assuming an angle between maximum pronation and supination, *ϕ_ps_*, of 60°, and the third value assumes *ϕ_ps_* equals 120°. We chose these angles mainly to demonstrate the magnitude of the p-s angle contribution to *L_N_*, but we estimate that the former angle might represent inclined stroke plane hovering whereas the latter might represent horizontal stroke plane hovering; the actual value of *ϕ_ps_* for this fly was not specified and was probably somewhere in between these values. While the difference between the first value of *L_N_* using only the flapping term (1.473) and those using the p-s term (1.467 at 60°; 1.449 at 120°) was not large, all are much lower than the standard *C_L_* value of 4.42 calculated by Ennos [7].

## Discussion

This paper does not offer any new explanation for how a wing generates lift, nor does it challenge use of the standard lift coefficient for fixed wings or its use for flapping wings along with a realistic estimation of the effective velocity. Instead, it proposes the application of the energy interpretation of the standard lift coefficient by adopting the total specific kinetic energy of the lifting system as the energy benchmark to compare lift generation by dissimilar lifting systems. Normalized lift equations for various lift-generating systems are presented in Table 1.

Two problems are seen when using the standard lift coefficient. First, when the standard *C_L_*—originally defined for fixed airplane wings—is applied to flapping wings, it must be heavily modified based on complex three-dimensional vectorial interactions of effective wing velocities and accompanying induced flow velocities with their corresponding simplifying flow field assumptions. Second, the maximum standard lift coefficient for a fixed wing without flaps in steady flow is around 1.5, yet for a rotating cylinder may exceed 9. This value implies that, at the same forward speed, a rotating cylinder generates nearly 6 times the maximum lift of a wing with the same planform area as the cylinder's cross section. These lift coefficient values are misleading, because the work done by the cylinder on the surrounding flow field while generating lift should be compared to the total specific kinetic energy possessed by the cylinder, and not by that of a static wing of the same planform area and forward speed.

The normalized lift is a non-dimensional lift parameter that evaluates the ability of a lifting surface to generate lift, and its usefulness depends on the inclusion of all important variables involved during the production of lift. We propose that an adequate lift parameter for a lifting surface should include the significant kinetic energy sources involved in the generation of lift and because kinetic energies are scalar in nature, these can be algebraically added together and used as the energy benchmark against which the work on the surrounding flow is measured.

In contrast to the standard lift coefficient, the normalized lift, *L_N_*, can be applied explicitly, without modification, even in very complicated situations, as long as all the sources of kinetic energy (e.g., forward speed, flapping frequency, rotational speed, flapping amplitude) affecting the generation of lift are considered. Normalized lift is derived from the work-energy theorem, and it explicitly connects a Lagrangian description, following the fluid elements, with an Eulerian description, following the flow past the object [16]. It does not require detailed measurements of the flow patterns and wakes around the lifting surface. It does not yield anomalously high values for animal flight or rotating cylinders, instead producing values in a range that allow direct comparison with fixed wings. Because it is focused on work and energy, the normalized lift is more appropriate for evaluating the costs of lift production of a large number of lifting systems. Finally and conveniently, the definition of the normalized lift is the same as the standard lift coefficient for fixed wings but can be expanded to other more complex lifting systems, and places all these on the same energy footing.

As described above, considering or neglecting a component of the specific kinetic energy available to the lifting surface, (e.g., the wing p-s during flapping), may give a slightly different normalized lift and so, the specific kinetic energies considered must be explicitly stated. The case of two rotating spheres moving through a fluid, one hollow and one solid, provides an instructive example of the difference between the standard lift coefficient and the normalized lift, and highlights the importance of explicitly stating the specific kinetic energies of the lifting surface considered in the normalized lift calculation. Assume that both spheres have the same diameter and mass, but the hollow sphere consists of a thin, dense shell whereas the solid sphere is made of an equal mass of a less dense material. At the same rotational and translational speeds, both spheres will have identical standard lift coefficients and will generate the same lift. Their normalized lift values, however, will not be the same. The hollow sphere will have a higher moment of inertia and hence, a higher total specific kinetic energy. Since both spheres are doing the same work on the fluid, the hollow sphere will have a slightly lower normalized lift. Consider this counterintuitive difference in the context of spinning spheres in Magnus effect in a wind tunnel: if accelerated by the same electric motor, the hollow sphere will require more energy to achieve the same angular velocity of the solid sphere, so in energy terms, the hollow sphere requires more energy input to get the same lift output. In other words, for the *same* energy input, the hollow sphere would produce less lift, which is reflected by its lower normalized lift. The apparently counterintuitive result of the normalized lift linked to the moment of inertia of the spheres is also reflected in the standard lift coefficient as it is linked to the specific kinetic energy of the airplane, namely, a higher specific kinetic energy will involve a lower lift coefficient.

Our second example, the long-tongued bat (*G. soricina*) in forward flight, demonstrates both very low and very high L_N_ values. We interpret the low *L_N_* (<0.4) to indicate that the bat is flying with a very low mean angle of attack and camber. In contrast, at 1.0 m s^−1^, the bat is nearly hovering and we calculate a very high mean *L_N_* of approximately 2.4. Although this *L_N_* value is higher than the *L_N_* value for the hovering long-eared bat (*P. auritus*) of the first example, the *G. soricina* specimens had more than 60% greater wing loading. Thus, *G. soricina* appears to require more aerodynamic enhancement (e.g., extreme angles of attack, possible vertical flow) than the hovering long-eared bat.

The normalized lift evaluates fixed, flapping and rotating lift devices by placing them on an equal energy footing, giving a more logical comparison of their ability to generate lift. This concept can be directly applied to the lift of rotating rotors and flapping wings, thrust of propellers, flapping wings of both animals and micro air vehicles, and undulating bodies and fins. One important benefit of the *L_N_* is that by casting the coefficient in work-energy terms, it provides a valuable index of the energetic cost of producing lift. As a corollary, the normalized lift shows that generating lift by means of flapping wings and rotating cylinders is not as cheap as may be implied by their standard lift coefficients.

## Supporting Information

Appendix S1 **List of Symbols Used in Manuscript.**
(DOC)

Appendix S2 **Normalized Lift Calculations for Flettner Rotor, Dorsoventral Flapping Movements, and Pronation-Supination.**
(DOC)

## References

[1] Anderson JD (2007). Fundamentals of Aerodynamics.

[2] Dudley R, Ellington CP (1990). Mechanics of forward flight in bumblebees. II. Quasisteady lift and power requirements.. Journal of Experimental Biology.

[3] Lentink D, Dickinson MH (2009). Biofluiddynamic scaling of flapping, spinning and translating fins and wings.. Journal of Experimental Biology.

[4] Halliday D, Resnick R (1970). Fundamentals of Physics.

[5] Lentink D, Muller UK, Stamhuis EJ, de Kat R, van Gestel W (2007). How swifts control their glide performance with morphing wings.. Nature.

[6] Muijres FT, Bowlin MS, Johansson LC, Hedenstrom A (2012). Vortex wake, downwash distribution, aerodynamic performance and wingbeat kinematics in slow-flying pied flycatchers.. Journal of the Royal Society Interface.

[7] Ennos AR (1989). The kinematics and aerodynamics of the free flight of some Diptera.. J Exp Biol.

[8] Norberg UM, Wu TY-T, Brokaw CJ, Brennan C (1975). Hovering flight in the pied flycatcher (*Ficedula hypoleuca*).. Swimming and Flying in Nature.

[9] Vogel S (1994). Life in moving fluids: the physical biology of flow.

[10] Reid EG (1924). Tests of rotating cylinders.. NACA Technical Reports.

[11] Augspurger CK (1986). Morphology and dispersal potential of wind-dispersed diaspores of neotropical trees.. American Journal of Botany.

[12] Hoerner SF, Borst HV (1975). Fluid-Dynamic Lift.

[13] Ellington CP (1984). The aerodynamics of hovering insect flight. I. The quasisteady analysis.. Philosophical Transactions of the Royal Society of London Series B: Biological Sciences.

[14] Wolf M, Johansson LC, von Busse R, Winter Y, Hedenstrom A (2010). Kinematics of flight and the relationship to the vortex wake of a Pallas' long tongued bat (Glossophaga soricina).. Journal of Experimental Biology.

[15] Nachtigall W, Rainey RC (1976). Wing movements and the generation of aerodynamic forces by some medium-sized insects.. Insect Flight.

[16] Fox RW, McDonald AT (1992). Introduction to Fluid Mechanics.

