# Supplementary material for: Normalized Lift: An Energy Interpretation of the Lift Coefficient Simplifies Comparisons of the Lifting Ability of Rotating and Flapping Surfaces
Source: PLoS One. 2012 May 21;7(5):e36732. doi: 10.1371/journal.pone.0036732 (PMC3357408; doi:10.1371/journal.pone.0036732)
Supplement: Appendix S1 — List of Symbols Used in Manuscript. (DOC) [file pone.0036732.s001.doc]

**Appendix S1. List of Symbols Used in Manuscript**

Symbol Description

*C_L_* Standard lift coefficient

*c_ave_* Mean wing chord

*d* Distance

*E* Kinetic energy

*e* Specific kinetic energy (kinetic energy per unit mass, *E/m*)

*F* Force

*f_f_* Flapping frequency

*f_ps_* Frequency of cyclic changes in wing incidence or pronation-supination

*I* Moment of inertia

*I*_x_ Moment of inertia of a wing about an axis parallel to the animal’s longitudinal body axis and through the wing articulation

*I­*_y_ Moment of inertia of a wing about the wing’s span-wise torsion axis

*L* Lift

*L_N_* Normalized lift

*m* Mass

p-s Pronation-supination

*r* Radius

*r_f_* Wing length from shoulder to tip (halfwing length); tip radius of a flapping wing

*S* Wing area

*u_nf_* Near field flow speed strongly determined by rotation or flapping

*u_T_* Tangential velocity

*v* Air speed

*v_h_* Horizontal component of glider’s air speed

*v_v_* Vertical component of glider’s air speed

*v*_∞_ Free stream speed, far field translational speed unaffected by rotation or flapping

*~~V~~* Volume

*ρ* Air density

*φ_f_* Flapping stroke amplitude

*φ_ps_* Maximum change in angle about a wing’s spanwise axis; amplitude of pronation-supination angle

*ω* Angular velocity

*ω_f ave_* Mean angular flapping velocity of a flapping wing, (2.*φ_f_. f_f_)/*57.3

*ω_ps ave_* Mean angular velocity about the pronation-supination axis, (2.*φ_ps_. f_ps_)/*57.3
