# Supplementary material for: Normalized Lift: An Energy Interpretation of the Lift Coefficient Simplifies Comparisons of the Lifting Ability of Rotating and Flapping Surfaces
Source: PLoS One. 2012 May 21;7(5):e36732. doi: 10.1371/journal.pone.0036732 (PMC3357408; doi:10.1371/journal.pone.0036732)
Supplement: Appendix S2 — Normalized Lift Calculations for Flettner Rotor, Dorsoventral Flapping Movements, and Pronation-Supination. (DOC) [file pone.0036732.s002.doc]

Appendix S2. Normalized Lift Calculations for Flettner Rotor, Dorsoventral Flapping Movements, and Pronation-Supination

Flettner Rotor

To calculate the normalized lift of a Flettner rotor (rotating cylinder in Magnus effect) with forward velocity *v* = *v_∞_* and angular velocity *ω*, we must first calculate the specific kinetic energy of the cylinder using eq. 9 from main text. A solid cylinder of radius *r* that rotates about its long axis has a moment of inertia *I* per unit mass, (*I/m*), which equals *½r^2^*, and its tangential velocity *u_T_* equals *ωr*. Substituting these values into equation 9 gives:

eq. S2.1

In the above equation *u_T_* is the tangential velocity of the cylinder. For convenience, we can write these terms as

The velocity *u_nf_* is the near-field velocity which equals (1/√2) *u_T_* for the rotating cylinder. The total specific kinetic energy for the rotating cylinder is thus *½v_∞_^2^ + ½(1/√2u_T_)^2^*.

Dorsoventral Flapping Movements (flapping while hovering)

To calculate the *L_N_* for a flapping flyer, we determine the total specific kinetic energy *e* due to flapping of the wings around the longitudinal x axis of the animal’s body. Based on the angular motion of the wing in its stroke plane, the *e* due to flapping is *½(I_x_/m)ω_f ave_^2^* where *I_x_* is the halfwing’s moment of inertia, and *ω_f ave_*  is the mean angular velocity of flapping. We treated the halfwing as a rectangular plate rotating about an axis parallel to the long axis of the animal’s body and passing through the wing articulation. The specific moment of inertia is thus *I_x_*/*m = ⅓r_f_^2^*, where *r_f_* is the wing length from the articulation to tip. Although this treatment ignores the wing’s taper and would seem to be an overestimate, in a comparison with 17 measured *I_x_* values from a variety of birds [17], the rectangle approximation averaged 95% of the measured *I_x_* values. Substituting the specific kinetic energies into the definition of *L_N_* (main-text equation 6) gives:

eq. S2.2

The average angular velocity due to flapping, *ω_f ave_* equals *2f_f_φ_f_*/57.3, where *f_f_* is the flapping frequency in beats per second and *φ_f_* is the stroke amplitude in degrees. Note that *v_∞_* = 0 as there is no forward speed because the animal is hovering.

Flapping Hovering with Pronation-Supination

The specific kinetic energy due to p-s movements is given by *e* = *½(I_y_/m)ω_ps ave_^2^*, where *I_y_* is the wing’s moment of inertia about its span-wise axis (*I_y_*/*m = c_ave_*/12, where *c_ave_* is the average wing chord), and *ω_ps ave_* is the wing’s mean angular velocity about its span-wise axis. Including this term in the summation in the denominator of equation 5 gives:

eq. S2.3

where the angular velocity *ω_ps ave_* equals 2*f_ps_φ_ps_*/57.3. Here, *f_ps_* is the cycle frequency of p-s changes (which is the same as the flapping frequency), and *φ_ps_* is the maximum angular excursion of the wing about its span-wise axis (i.e., the angle between maximum pronation and maximum supination: the amplitude of the pronation-supination excursion).
